# Human Case of Severe Fever with Thrombocytopenia Syndrome Virus Infection, Taiwan, 2019

**DOI:** 10.3201/eid2607.200104

**Published:** 2020-07

**Authors:** Shih-Huan Peng, Su-Lin Yang, Shih-En Tang, Tzy-Chen Wang, Tung-Chien Hsu, Chien-Ling Su, Meng-Yu Chen, Masayuki Shimojima, Tomoki Yoshikawa, Pei-Yun Shu

**Affiliations:** Centers for Disease Control, Ministry of Health and Welfare, Taipei, Taiwan (S.-H. Peng, S.-L. Yang, T.-C. Wang, T.-C. Hsu, C.-L. Su, M.-Y. Chen, P.-Y. Shu);; Tri-Service General Hospital, Taipei (S.-E. Tang);; National Defense Medical Center, Taipei (S.-E. Tang);; Institute of Aerospace and Undersea Medicine, National Defense Medical Center, Taipei (S.-E. Tang);; National Institute of Infectious Diseases, Tokyo, Japan (M. Shimojima, T. Yoshikawa)

**Keywords:** severe fever with thrombocytopenia syndrome, autochthonous, severe fever with thrombocytopenia syndrome virus, Taiwan, viruses, Huaiyangshan banyangvirus, thrombocytopenia, case study, clinical findings, Pseudomonas aeruginosa, bacteria, co-infection

## Abstract

We report on a 70-year-old man with fever, leukopenia, thrombocytopenia, vomiting, malaise, dyspnea, and consciousness disturbance who was infected with severe fever with thrombocytopenia syndrome virus in northern Taiwan, 2019. This autochthonous case was confirmed by reverse transcription PCR, virus isolation, and genomic sequencing.

Severe fever with thrombocytopenia syndrome (SFTS) is a tickborne infection caused by the SFTS virus (SFTSV, also known as *Huaiyangshan banyangvirus*), which was identified in China in 2009 (*1*) and afterward in South Korea (*2*), Japan (*3*), and Vietnam (*4*). Since then, the number of SFTS cases in East Asia has risen rapidly. Therefore, laboratory-based surveillance of SFTS has been conducted in the routine molecular diagnosis of arboviral infections in the Taiwan Centers for Disease Control (Taiwan CDC) since 2013. We identified a patient in Taiwan with laboratory-confirmed SFTS who was originally suspected of having dengue or rickettsial infections.

In November 2019, a 70-year-old man who lived in northern Taiwan and had no travel history was admitted to the hospital with a 9-day history of fever (38.8°C–39.2°C), chills, nausea, vomiting, and malaise. The patient had underlying hypertension and type 2 diabetes mellitus that was controlled without medication. At hospital admission, we noted a generalized rash over the trunk and both feet. Laboratory examinations showed that the patient had leukopenia; thrombocytopenia; abnormal prothrombin time; elevated levels of aspartate transaminase, alanine transaminase, creatinine kinase, and C-reactive protein; and diagnostic disseminated intravascular coagulation (Table). Chest radiography and chest computed tomography showed patchy consolidations and ground-glass opacities of both lungs. A few hours after admission, the patient experienced a general tonic–clonic seizure, with worsening consciousness and dyspnea. He was transferred to the intensive care unit, where intubation and ventilator support began. He also received massive blood transfusions for severe thrombocytopenia, active mucosal (oral, nasal, and gastrointestinal tract) bleeding, and disseminated intravascular coagulation. Blood and sputum cultures revealed that the patient was infected with *Pseudomonas aeruginosa*; he received piperacillin/tazobactam, doxycycline, and clarithromycin as empirical therapy. Results of laboratory tests for hepatitis A and B viruses, cytomegalovirus, herpes simplex virus, adenovirus, and influenza were all negative. After the patient received a diagnosis of SFTSV infection, he received treatment with intravenous immunoglobulin for 5 days. However, his condition continued to deteriorate progressively. The patient died on day 40 after illness onset as a result of multiorgan failure. Delayed diagnosis and the presence of underlying conditions in this patient, including hypertension and diabetes mellitus, may be associated with his severe disease and death (*5*).

**Table:** Laboratory findings of patient with severe fever with thrombocytopenia syndrome virus infection, Taiwan, 2019.

Laboratory finding	Patient value	Reference range
Leukocytes, cells/μL	1.550 × 10^3^	3.9–10.6 × 10^3^
Erythrocytes, cells/μL	5.550 × 10^6^	3.9–5.4 × 10^6^
Hemoglobin, g/dL	16.0	12–16
Platelets/μL	41 × 10^3^	150–400 × 10^3^
% Neutrophils	68.4	42–74
% Lymphocytes	29.7	25–56
Aspartate transaminase, U/L	1,326	0–37
Alanine transaminase, U/L	569	0–40
Creatinine kinase, U/L	1,310	56–224
Creatinine, mg/dL	1.7	0.44–1.03
C-reactive protein, mg/L	7.8	<5
Total bilirubin, mg/dL	1.7	0.2–1.2
Glucose, mg/dL	250	70–100
Prothrombin time, s	14.4	6.6–11.6
Activated partial thromboplastin time, s	63.4	23.9–34.9

The patient often spent time on a vegetable farm in a mountainous area without wearing shoes, raising suspicions for arboviral and rickettsial infections. The hospital sent blood samples, collected from the patient before the blood transfusions on day 12 after illness onset, to the Taiwan CDC for laboratory diagnosis of arboviral and rickettsial diseases. Arboviral infections were detected using primer sets (Appendix Table 1) by SYBR-Green I-based real-time reverse transcription PCR (RT-PCR). In addition, we detected the SFTSV genome using SFTSV-specific primer sets targeting nonstructural protein and nucleocapsid protein genes. Results of RT-PCR and PCR for flavivirus and chikungunya virus infections, scrub typhus, murine typhus, spotted fever rickettsiae, and leptospirosis were all negative. 

SFTSV was isolated from patient serum with the Vero cell line and confirmed by RT-PCR and immunofluorescence assays. SFTSV RNA remained undetected in the urine sample. The viral loads in serum continuously decreased from day 12 after disease onset and became undetectable on day 29 after disease onset (Appendix Figure 1). SuperScript III 1-step RT-PCR (http://www.thermofisher.com) identified partial small (S), medium (M), and large (L) segments of SFTSV in the serum collected on day 12 after disease onset using a different set of primers (Appendix Table 2). SFTSV has been classified into 6 different genotypes according to its genome sequence (*6*). Phylogenetic analyses of the partial S (1,704 bp; GenBank accession no. MN830173), M (3,340 bp; GenBank accession no. MN830174), and L (6,332 bp; GenBank accession no. MN910270) segment sequences using MEGA7 (*7*) using the maximum-likelihood method (Appendix Figures 2, 3) showed that the partial S, M, and L segments of SFTSV from this patient belong to genotype B and are closely related to Japanese strains. SFTSV identified in *Rhipicephalus microplus* ticks in central Taiwan belongs to genotype A/C (*8*).

All of the patient’s close contacts, including 8 family members, a friend, and 60 medical personnel, were healthy and without symptoms during the monitoring period. Six mites from 2 brown rats (*Rattus norvegicus*) and 2 Asian house shrews (*Suncus murinus*) were captured in the area surrounding the residence of the patient. All the RNA samples from animals and mites showed SFTSV negative results by RT-PCR.

Although the main SFTSV tick vector, *Haemaphysalis longicornis*, has not been documented in Taiwan, other tick vectors, such as *R. microplus* and *Amblyomma testudinarium*, have been found in wild and domestic animals (*8*–*10*). Further studies on the identification of natural vectors and routes of transmission are needed. The presence of an emerging SFTS case highlights the need for further studies of the prevalence, geographic distribution, and surveillance of SFTSV in Taiwan.

Appendix Additional information on human case of severe fever with thrombocytopenia syndrome virus infection, Taiwan, 2019.
