# Supplementary material for: Human Case of Severe Fever with Thrombocytopenia Syndrome Virus Infection, Taiwan, 2019
Source: Emerg Infect Dis. 2020 Jul;26(7):1612–4. doi: 10.3201/eid2607.200104 (PMC7323535; doi:10.3201/eid2607.200104)
Supplement: Appendix — Additional information on human case of severe fever with thrombocytopenia syndrome virus infection, Taiwan, 2019. [file 20-0104-Techapp-s1.pdf]

Article DOI: https://doi.org/10.3201/eid2607.200104

Human Case of Severe Fever with Thrombocytopenia Syndrome Virus Infection, Taiwan, 2019

Appendix

RNA Extraction and Reverse Transcription PCR (RT-PCR)

We extracted RNA from 140 μL acute-phase sera specimens using the QIAamp Viral RNA Mini Kit (QIAGEN, https://www.qiagen.com) according to the manufacturer's instructions. We performed real-time RT-PCR amplification using Quantitect SYBR green RT PCR kit (QIAGEN) with the following parameters: 50°C for 30 min; 95°C for 15 min and 45 cycles of 95°C for 15 sec, 55°C for 30 sec, 72°C for 20 sec, and 77°C for 20 sec; and 95°C for 1 min, melting curve program from 68°C to 95°C. We analyzed the results using the software program of the LightCycler 96 or 480 Real-Time PCR system (Roche Diagnostics, https://www.roche.com). The plasmid containing S genomic segment of severe fever with thrombocytopenia syndrome virus (SFTSV) HB29 strain (GenBank accession no. NC 018137) served as positive control for the RT-PCR assay.

RT-PCR and Nucleotide Sequencing

We extracted RNA from the patient's serum sample and further subjected it to RT-PCR for sequencing. The primers were designed based on the S (AB985557), M (AB985653 and AB985320), and L (AB983531) segment sequences. We performed RT-PCR using Superscript III One-Step RT-PCR system with Platinum Taq High Fidelity (Invitrogen, https://www.thermofisher.com) under the following conditions: 55°C for 30 min; 94°C for 2 min and 40 cycles of 94°C for 15 sec, 50°C or 54°C for 30 sec, and 68°C for 1 min; and a prolonged extension at 68°C for 5 min. We sequenced the RT-PCR products directly by using the BigDye Terminator Cycle Sequencing Kit and the ABI 3730xl DNA analyzer (Applied Biosystems,

https://www.thermofisher.com) according to the manufacturer's protocol. We used each forward and reverse primer for sequencing and the overlapping sequences were combined.

References

1. Zhang YZ, He YW, Dai YA, Xiong Y, Zheng H, Zhou DJ, et al. Hemorrhagic fever caused by a novel Bunyavirus in China: pathogenesis and correlates of fatal outcome. Clin Infect Dis. 2012;54:527–33. PubMed http://dx.doi.org/10.1093/cid/cir804

2. Yoshikawa T, Fukushi S, Tani H, Fukuma A, Taniguchi S, Toda S, et al. Sensitive and specific PCR systems for detection of both Chinese and Japanese severe fever with thrombocytopenia syndrome virus strains and prediction of patient survival based on viral load. J Clin Microbiol. 2014;52:3325–33. PubMed http://dx.doi.org/10.1128/JCM.00742-14

3. Sun Y, Liang M, Qu J, Jin C, Zhang Q, Li J, et al. Early diagnosis of novel SFTS bunyavirus infection by quantitative real-time RT-PCR assay. J Clin Virol. 2012;53:48–53. PubMed http://dx.doi.org/10.1016/j.jcv.2011.09.031

4. Shu PY, Chang SF, Kuo YC, Yueh YY, Chien LJ, Sue CL, et al. Development of group- and serotype-specific one-step SYBR green I-based real-time reverse transcription-PCR assay for dengue virus. J Clin Microbiol. 2003;41:2408–16. PubMed http://dx.doi.org/10.1128/JCM.41.6.2408-2416.2003

<jrn>5. Pastorino B, Bessaud M, Grandadam M, Murri S, Tolou HJ, Peyrefitte CN. Development of a TaqMan RT-PCR assay without RNA extraction step for the detection and quantification of African chikungunya viruses. J Virol Methods. 2005;124:65–71. PubMed http://dx.doi.org/10.1016/j.jviromet.2004.11.002

6. Wen HL, Zhao L, Zhai S, Chi Y, Cui F, Wang D, et al. Severe fever with thrombocytopenia syndrome, Shandong Province, China, 2011. Emerg Infect Dis. 2014;20:1–5. PubMed http://dx.doi.org/10.3201/eid2001.120532

Appendix Table 1. Primers used for SYBR green I-based real-time RT-PCR assay for severe fever with thrombocytopenia syndrome virus infection, Taiwan, 2019.*

Name	Sequence (5’ to 3’)	Amplicon (bp)	Reference
SFTSV-SF	ACCTCTTTGACCCTGAGTTWGACA	125	(*1*)
SFTSV-SR	CTGAAGGAGACAGGTGGAGATGA		
SFTSV-NP-1F	ATCGTCAAGGCATCAGGGAA	458	(*2*)
SFTSV-NP-1R	TTCAGCCACTTCACCCGAA		
SFTSV-NP-2F	CATCATTGTCTTTGCCCTGA	461	(*2*)
SFTSV-NP-2R	AGAAGACAGAGTTCACAGCA		
SFTS-1F	GGAAACTGGRAGAGAGAACT	240	This study
SFTS-1R	GAAGTGAACAAGTGGTGGTT		
SFTSV-LF	AGTCTAGGTCATCTGATCCGTTYAG	92	(*3*)
SFTSV-LR	TGTAACTTCGCCCTTTGTCCAT		
DN-F	CAATATGCTGAAACGCGAGAGAAA	171	(*4*)
DN-R1	CCCCATCTAACCAATATTCCTGCT		
DN-R2	CCCCATCTGTTCAGTATCCCTGCT		
FL-F1	TGYGTBTACAACATGATGGG	272 or 200	This study
FL-F2	ATATGGTACATGTGGCTAGGAGC		
FL-R	GTGTCCCANCCHGCTGTGTCA		
F-CHIK	AAGCTYCGCGTCCTTTACCAAG	209	(*5*)
R-CHIK	CCAAATTGTCCYGGTCTTCTT		

*RT-PCR, reverse transcription PCR; SFTSV, severe fever with thrombocytopenia syndrome virus.

Appendix Table 2. Primers used for conventional RT-PCR assay and sequencing for severe fever with thrombocytopenia syndrome virus infection, Taiwan, 2019.*

Name	Sequence (5' to 3')	Amplicon (bp)	Reference
SFTSVS1_F	ACACAAAGAACCCCCAAAAAAG	605	This study
SFTSVS1_R	CTCCGCGCATCTTCACATTG		
SFTSVS2_F	GTCAGAGTGGTCCGGAGTTG	560	
SFTSVS2_R	CTCCGCGCATCTTCACATTG		
SFTSVS3_F	GGTTGTGGAATGGGCACCTA	730	
SFTSVS3_R	ACTGTCAGGCTTGAGCCATC		
SFTSVS4_F	CTGATTCCAACTGCAGGGGT	747	
SFTSVS4_R	GCCATAGAGAAGCGAAGGCT		
SFTSVS5_F	AGCCTTCGCTTCTCTATGGC	523	
SFTSVS5_R	ACACAAAGAACCCCCTTCAT		
SFTSVM1_F	ACACAGAGACGGCCAACA	774	
SFTSVM1_R	GATGAGCTCTCTTGCACCCA		
SFTSVM2_F	ACCTCAGCCCCTTGATGTTG	690	
SFTSVM2_R	TTTGCAGGGTAGCACTGAGG		
SFTSVM3_F	GTGGCAAGAAAAGCACGGAG	660	
SFTSVM3_R	GCAGACATGCCTCTTGTCCT		
SFTSVM4_F	TCTGTGAGCTGCTTGATGGG	836	
SFTSVM4_R	TCACACAGGTCAGTCAAGCC		
SFTSVM5_F	GGCCCCTTCATGCATCTTCT	746	
SFTSVM5_R	ACGCAGTCTCATCCCCTTTG		
SFTSVM6_F	CCCTTGGACATCACAGCCAT	640	
SFTSVM6_R	CAACTCCCCTGAGAGCACTG		
SFTSVM7_F	CCCTTGGACATCACAGCCAT	723	
SFTSVM7_R	ACACAAAGACCGGCCAACAC		
SFTSVL1_F	ACACAGAGACGCCCAG	857	
SFTSVL1_R	GATCAGCATAGGCCTCCACC		
SFTSVL2_F	CCAGGCCTGTACGACCAAAT	834	
SFTSVL2_R	CTCTAGCCGCTCCTGAATGG		
SFTSVL3_F	CCATTCAGGAGCGGCTAGAG	901	
SFTSVL3_R	TCCCCAGCATCAATGGTGTC		
SFTSVL4_F	CATCTGGATTGCATGGTGCG	728	
SFTSVL4_R	GGGCTCAACTCACAGACACA		
SFTSVL5_F	CAGAACCTTGAGGAGCGTGT	886	
SFTSVL5_R	TGACATCCACCACCCCATTG		
SFTSVL6_F	CAATGGGGTGGTGGATGTCA	748	
SFTSVL6_R	CTGCTCCACCCAGTCTTCAG		
SFTSVL7_F	CCTCATGGACAACCCTGCAT	856	
SFTSVL7_R	AAAGGGGCCATCCCTCAATG		
SFTSVL8_F	AGGTGTGGTTTGGCCTGAAA	857	
SFTSVL8_R	ACAGCCGTCAGTCCTTGATG		
SFTSVL9_F	TGCATGGTTAGGCTGAGTGG	997	
SFTSVL9_R	ACACAAAGACCGCCCAGATC		
SFTSV_1324LF	GGCAGCAAACCAGAAGAAAG	1003	(*6*)
SFTSV_2326LR	CATTTCTCCGAGGGCATTTA		

*RT-PCR, reverse transcription PCR; SFTSV, severe fever with thrombocytopenia syndrome virus.

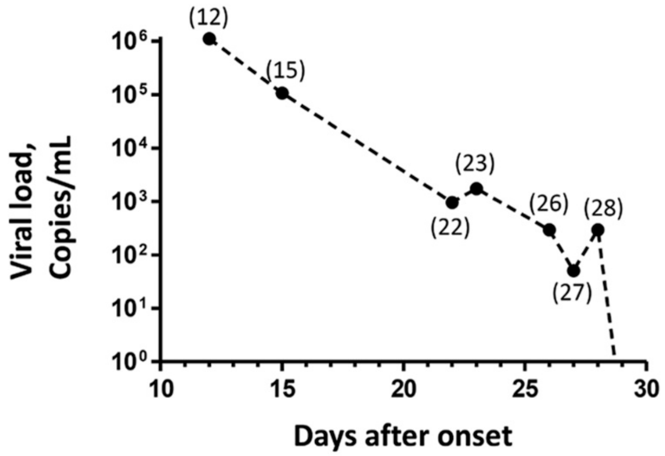

Appendix Figure 1. Viral loads in serum samples of the severe fever with thrombocytopenia syndrome case in Taiwan. Viral copy numbers were calculated by SYBR-Green I-based real-time one-step RT-PCR with an SFTSV-specific primer set (SFTSV-SF and SFTSV-SR). The days after disease onset are marked.

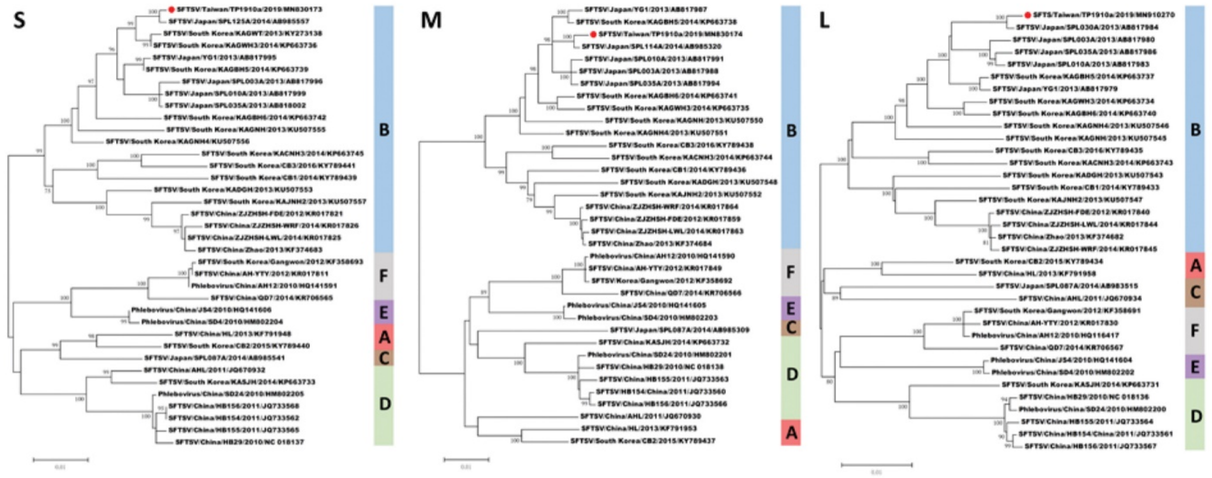

Appendix Figure 2. Phylogenetic analysis of severe fever with thrombocytopenia syndrome case in Taiwan. Trees show the genetic relationships of six genotypes according to the partial S segment (1,704 bp) containing the complete nucleocapsid and nonstructural protein gene sequences of SFTSV strains (S), the partial M segment (3,340) containing the complete membrane glycoprotein gene sequence of SFTSV strains (M) and the partial L segment (6,332 bp) containing the complete RNA polymerase gene sequence of SFTSV strains (L). Viruses were identified by virus/country/strain/year of isolation/GenBank accession no. The analysis was performed by using MEGA7 software and the maximal-likelihood method (Kimura 2-parameter model). Bootstrap support values ≥ 75 are shown (1,000 replicates) along the branches. Scale bars indicate nucleotide substitutions per site.

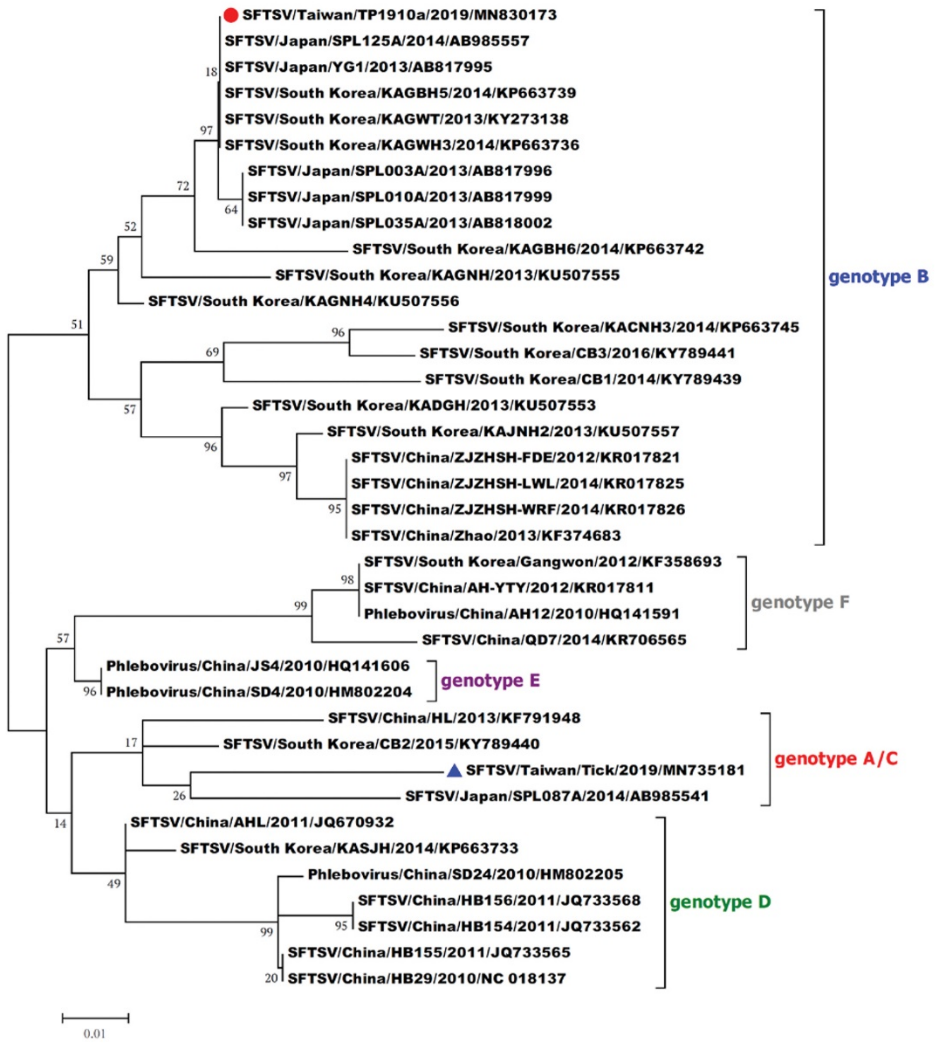

Appendix Figure 3. Phylogenetic analysis of severe fever with thrombocytopenia syndrome virus identified in the human case (red solid circle) and *R. microplus* tick (blue solid triangle) in Taiwan. Trees show the genetic relationships of six genotypes according to the partial S segment nucleotide sequences (304 bp) of nucleocapsid protein gene of SFTSV strains. Viruses were identified by virus/country/strain/year of isolation/GenBank accession no. The analysis was performed by using MEGA7 software and the maximal-likelihood method (Kimura 2-parameter model). All bootstrap support values are shown (1,000 replicates) along the branches. Scale bars indicate nucleotide substitutions per site.
